# Current Hospital Topics

**Published:** 1907-02-16

**Authors:** 


### HOSPITAL ADMINISTRATION.

### CONSTRUCTION AND ECONOMICS.

### CURRENT HOSPITAL TOPICS.

#### Street Ambulances in London.

THE proceedings at the Conference at Gray's Inn on Saturday, an account of which we publish this week, afford the most conclusive evidence of the wisdom of the Home Secretary in appointing a departmental committee to consider the existing provision for street accidents in London. An examination of the various forms of ambulance exhibited on Saturday, being those at present in use, coupled with the fact that the best counsel that the majority of the conference could give was to welcome as adequate to the necessities of the case the addition of 70 police litters to those now available, proves to demonstration that the promoters of that conference are out of touch with public sentiment in this matter. What London needs is one well organised, carefully-thought-out and adequate system dealing with all street accidents, whenever they may occur, in any part of London. We hope, therefore, that the departmental committee, after taking ample evidence, may report to the Home Secretary in favour of such a system and its introduction by the County Council without a moment's avoidable delay. We have always regretted that Mr. H. L. Bischoffsheim's splendid generosity in bearing the whole cost of the existing system of street ambulances for 18 years has met with so little recognition. That system must form the basis on which the new organisation will rest, and Mr. Bischoffsheim's generosity is likely to reduce to a minimum the cost to the ratepayers of a new and perfected system. All honour to Mr. Bischoffsheim and those who have worked with him in lessening the suffering of every Londoner who meets with an accident in the streets of the metropolis.

#### The Northampton County Hospital.

THE 163rd annual report of this institution is highly creditable to the committee and the secretary-superintendent. It is well written, well printed, full of interesting points, and shows clearly the progress made and the requirements yet to be fulfilled. The information given of the findings of a special committee on the domestic department of the hospital is important. The question of English and foreign meat is dealt with, and although the latter is shown to be cheaper, it has been decided to purchase English meat by preference for the whole establishment. We called attention to the water question dealt with in this report some months ago. Whilst we agree that every effort should be made to prevent waste, no hospital can be considered well administered which has not an abundant supply of water, and also regulations which afford the maximum of facilities for the use of baths by the staff and the patients. Several hospitals have recently appointed economy committees, and the work of such a committee, apart from its interest, which is considerable, is shown by the fact that at Northampton the direct savings expected to result from the inquiry are at least £300 in the first year, with greater saving in subsequent years. The committee take credit for having introduced the uniform system of accounts, which led to the discovery that there was great waste in the housekeeping department, and so the sub-committee just referred to was appointed. The report contains some illustrations which exhibit the special value of the roomy balconies and of the extensive views they command. There is, further, an excellent illustration of one of the new wards, which will show the expert how exceedingly good and up-to-date these wards are. The Northampton County Hospital, as reconstructed and perfected, may claim to be one of the best, if not the best, of county hospitals. It would facilitate reference if a list of contents were to be included in future reports.

#### Hospital Accounts and the Financial Year.

THE uniform system of accounts which has recently been overhauled and amplified by a commitee of hospital secretaries, under the advice and with the assistance of Mr. J. G. Griffiths, now constitutes the system upon which the accounts of all the best administered British hospitals are kept. The King's Hospital Fund have experienced the difficulty, which all who have to do with hospital statistics come to realise sooner or later, that it is essential that the hospital year of each institution shall end on the same date. The majority of hospitals, like the majority of businesses and private individuals, make up their accounts for the year ending December 31st. There are, however, still some old-fashioned instituions where the financial year is made to end on some other day—*i.e.*, March 31st or June 30th, or September 30th, or even May 31st or October 31st. The King's Fund have now laid down a rule that all hospitals seeking a grant must close their accounts to December 31st in each year. We hope that hospital committees throughout the country will follow this rule, and, where necessary, give instructions that this shall be the date on which the financial year at their institution must, in future, end. In no other way is it possible for the managers of a large hospital readily to compare its expenditure and working with that of other similar institutions. The new edition of "Burdett's Hospitals and Charities" contains an intimation that in future volumes only those hospitals which make up their accounts to December 31st in each year can be included in the chapters and tables dealing with hospital income and expenditure. Under this new scheme every one using this book will have, in the tables in question, a reliable list of the most up-to-date hospitals, which are managed on business principles. There has been some competition in the past to secure a place in these chapters, and that competition is likely to become more keen in consequence of the new rule, which should make inclusion even more valuable in the future than it has been in the past.

